# Temporal and spatial adaptation of transient responses to local features

**DOI:** 10.3389/fncir.2012.00074

**Published:** 2012-10-18

**Authors:** David C. O'Carroll, Paul D. Barnett, Karin Nordström

**Affiliations:** ^1^Adelaide Centre for Neuroscience Research, School of Medical Sciences, The University of AdelaideAdelaide, SA, Australia; ^2^Department of Neuroscience, Uppsala UniversityUppsala, Sweden

**Keywords:** salient feature, EMD, motion detection, motion adaptation, insect vision, spatial integration, local contrast sensitivity

## Abstract

Interpreting visual motion within the natural environment is a challenging task, particularly considering that natural scenes vary enormously in brightness, contrast and spatial structure. The performance of current models for the detection of self-generated optic flow depends critically on these very parameters, but despite this, animals manage to successfully navigate within a broad range of scenes. Within global scenes local areas with more salient features are common. Recent work has highlighted the influence that local, salient features have on the encoding of optic flow, but it has been difficult to quantify how local transient responses affect responses to subsequent features and thus contribute to the global neural response. To investigate this in more detail we used experimenter-designed stimuli and recorded intracellularly from motion-sensitive neurons. We limited the stimulus to a small vertically elongated strip, to investigate local and global neural responses to pairs of local “doublet” features that were designed to interact with each other in the temporal and spatial domain. We show that the passage of a high-contrast doublet feature produces a complex transient response from local motion detectors consistent with predictions of a simple computational model. In the neuron, the passage of a high-contrast feature induces a local reduction in responses to subsequent low-contrast features. However, this neural contrast gain reduction appears to be recruited only when features stretch vertically (i.e., orthogonal to the direction of motion) across at least several aligned neighboring ommatidia. Horizontal displacement of the components of elongated features abolishes the local adaptation effect. It is thus likely that features in natural scenes with vertically aligned edges, such as tree trunks, recruit the greatest amount of response suppression. This property could emphasize the local responses to such features vs. those in nearby texture within the scene.

## Introduction

As animals move through the natural surround their progress generates wide-field optic flow across the retina. Behaviorally generated optic flow is used to visually guide behavior in both vertebrates and invertebrates (e.g., Warren and Rushton, 2009; Srinivasan, 2011). Flying animals may use optic flow cues to e.g., maintain an intended flight path or a hovering stance, and to avoid obstacles (e.g., Tammero and Dickinson, 2002; Reiser and Dickinson, 2010; de Vries and Clandinin, 2012). For some of these visually guided behaviors, the location of salient features within the scene is also relevant. Indeed, it has been shown that many animals, vertebrates as well as invertebrates, visually orient toward salient features (Götz, 1975; Caduff and Timpf, 2008; Maimon et al., 2008; Sareen et al., 2011).

There is broad evidence that most animals with eyes compute local motion in a fundamentally similar way (see e.g., Borst and Euler, 2011) using a spatio-temporal correlation of the luminance change from two neighboring inputs associated with a moving stimulus. In flies, optic flow is analyzed in lobula plate tangential cells (LPTCs) by spatially pooling inputs from large arrays of local elementary motion detectors (EMDs) (see Borst et al., 2010). LPTCs have been shown to be involved in behavioral responses to visual motion (Heisenberg et al., 1978; Geiger and Nässel, 1981; Hausen and Wehrhahn, 1990) and have complex receptive fields that support an important role in visually guided navigation (Krapp and Hengstenberg, 1996).

The detection of wide-field motion has generally been studied using relatively uniform stimuli, such as sinusoidally modulated gratings (see e.g., Clifford and Ibbotson, 2002). When stimulated with these simple stimuli, LPTCs show a dependence on pattern contrast and spatial frequency well predicted by simple computational models for the EMD (Borst et al., 2010). However, natural scenes are often much more complex, containing numerous high-contrast local features such as tree trunks, borders between the horizon and the sky, or other sharp boundaries between shaded and well illuminated areas, in addition to lower-contrast “inner texture.” Indeed, recent studies have suggested that the dynamic non-linear properties of visual neurons are likely to be optimized for the statistics of natural signals (Schwartz and Simoncelli, 2001).

Recent work has highlighted that neural responses to natural scenes are strongly influenced by the spatio-temporal distribution of features within it (Meyer et al., 2011; Liang et al., 2012). We recently showed that LPTCs adapt differentially to natural scenes in a manner that improves reliability for velocity coding (Barnett et al., 2010). We have also shown that vertically elongated features in natural scenes affect the global responses of LPTCs to a greater degree than would be suggested by the receptive field properties alone (O'Carroll et al., 2011). Such features would generate potent local transient responses from EMDs, but little is known about how dynamic adaptation to local stimuli affects subsequent responses. Ideally, such experiments should be carried out by recording responses to natural scenes from the EMDs themselves. Whereas the evidence for retinotopic EMD-like elements as the inputs to LPTCs is overwhelming (Borst and Euler, 2011), electrophysiological recordings from such retinotopic neurons have proved elusive. However, recording from LPTCs is feasible and reliable. In such recordings the gain reduction component of motion adaptation has been shown to be very local, likely operating at the level of individual EMDs (Maddess and Laughlin, 1985; Neri and Laughlin, 2005; Kurtz et al., 2009; Nordström and O'Carroll, 2009). Furthermore, it operates on a rapid time-scale comparable to the low-pass filters inherent to the motion detectors themselves (Nordström et al., 2011). Passage of a high-contrast feature might thus induce sufficient local adaptation to significantly reduce responses to subsequent features passing the same point in space. This could affect not only the global response to a scene as coded by LPTCs, but also the relative salience of features analyzed by other neuronal pathways (e.g., for feature discrimination) taking input from the same local EMDs.

Studying this phenomenon is complicated when using fully naturalistic stimuli, however. As stimuli contain increasing numbers of local features, varying spatial frequencies and local contrasts, experiments get more difficult to control and the data harder to interpret because of the difficulty in associating global responses with specific local features or feature clusters. To be able to more conclusively quantify the effect of local features, we therefore use experimenter-designed stimuli to determine how the spatio-temporal distribution of local features interacts with each other to influence the response of LPTCs. This provides us with precise control over the temporal and spatial characteristics of the stimuli, to enable direct correlation between specific image features and neural response. We recorded intracellularly from HS neurons, which respond with graded membrane potential changes, making it possible to record responses that would otherwise be below the spike threshold. We show that local high-contrast features recruit powerful local adaptation and suppress the response to subsequently seen features. By varying the distribution of the stimuli, we show that this local gain reduction is facilitated by simultaneous activation of neighboring motion sensitive elements. These two effects may combine to enhance the salience of “dominant” high-contrast features within scenes, such as vertically oriented boundaries.

## Materials and methods

### Electrophysiology

We used wild caught hoverflies, *Eristalis tenax*, immobilized with wax and mounted 14–15 cm in front of a CRT display. We performed sharp electrode intracellular recordings on Horizontal System (HS) neurons in the left lobula plate using aluminosilicate electrodes pulled on a Sutter Instruments P97 electrode puller with a 3 × 3 mm box filament. Electrodes were filled with 2 M KCl and typically had tip resistances of 80–250 MΩ. Each neuron was identified based on its distinctive receptive field as characterized in our earlier work (Nordström et al., 2008).

### Data acquisition and analysis

Data were digitized at 5 kHz using a 16-bit A/D converter (National Instruments, Austin Texas, United States) and analyzed off-line with Matlab (http://www.mathworks.com). In all experiments, we normalized the membrane potential by subtracting the average resting membrane potential recorded for 1 s immediately prior to each trial. HS neurons display activity-induced spikelets, thereby adding an additional non-linearity to the axonally recorded membrane potential (Hengstenberg, 1977; Haag et al., 1997). To reduce the influence of such spikelets in our analysis, we spike filtered our data by removing spike-like events and replacing them with the local mean membrane potential (see Nordström and O'Carroll, 2009). To quantify response differences we averaged the membrane potential in a time window that coincided with the stimulus.

All statistics were performed using GraphPad Prism (http://www.graphpad.com). *N* refers to the number of animals, and *n* refers to the total number of repetitions across neurons. All data are presented as mean ± standard error of the mean (SEM), where the mean and statistics are computed across animals (*N*), unless otherwise mentioned.

### Stimuli

**Figure 1:**
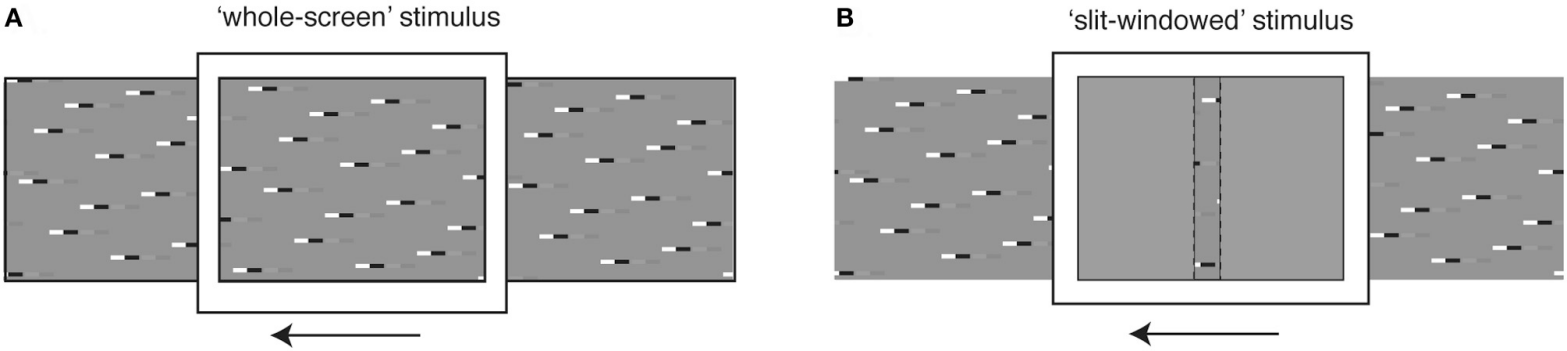
**Stimulus display modes. (A)** The *whole screen mode* is designed to stimulate large regions of the neuron's receptive field simultaneously, to investigate global response properties. **(B)** The *slit windowed mode* limits the stimulus width horizontally, to enable measurement of local response properties. The slit is highlighted with a dashed line for illustration purposes. During experiments there was no border between the slit and the mean-luminance background.

Panoramic stimulus images comprising various combinations of square-wave “doublet” features were computed in Matlab and displayed on a linearized, 8-bit, RGB CRT at 200 Hz refresh rate and with a mean luminance of 100 Cd/m^2^ using VisionEgg software (Straw, 2008). Textures were animated either via the entire screen (Figure 1A), which subtended approximately 100 × 75° of the hoverfly's visual field, or via a “slit-windowed” stimulus (Figure 1B) that masked the width of the viewport onto the pattern to 2.5° wide, corresponding to only a few ommatidia in the fronto-dorsal visual field (Straw et al., 2006). The remaining field of the CRT monitor was filled with uniform mid-gray.

### Model predictions

We used an elaborated Hassenstein—Reichardt correlator model to predict local motion detector responses to doublet features. This model incorporated spatial and temporal filtering processes matched to the optics, early vision, and motion computation of LPTCs in the hoverfly *Eristalis tenax* (Dror et al., 2001; Straw et al., 2008; O'Carroll et al., 2011). The model uses a linear subtraction of the half-units, so that responses to preferred and anti-preferred direction motion are perfectly mirror symmetrical. Full details of this basic EMD model are given in O'Carroll et al. (2011).

Our slit-windowed stimulus (Figure 1B) would not only stimulate the “central” local motion detectors contributing to the receptive field of the HS neurons (i.e., those with receptive fields corresponding to the center of the 2.5° slit mask). In addition, the neural response would receive contributions from adjacent local motion detectors, whose receptive fields extend beyond the mask, and which would only be partially stimulated via one “input arm,” We therefore simulated the masked stimulus via a full array of EMDs, with an inter-detector spacing of 1.1° and a Gaussian blur of 1.4° half-width on the inputs. The model thus accounts for effects caused by partial stimulation of EMDs at the edges of the slit-windowed mask.

## Results

### Response characteristics of local motion-sensitive elements

To determine the influence of local features on the global response, we used two stimulus display modes. The *whole-screen* mode (Figure 1A) displays the stimulus across the width of the monitor, thus allowing us to investigate neural responses following spatial integration within the HS receptive field. The *slit-windowed* mode (adapted from Reichardt and Egelhaaf, 1988; Egelhaaf et al., 1989) limits the width of the stimulus to the size of a few local EMDs (2.5° wide, Figure 1B). This means that only a fraction of the image is seen at any one point in time and the response we record reflects the output of local motion elements in a small region of the visual field.

**Figure 2:**
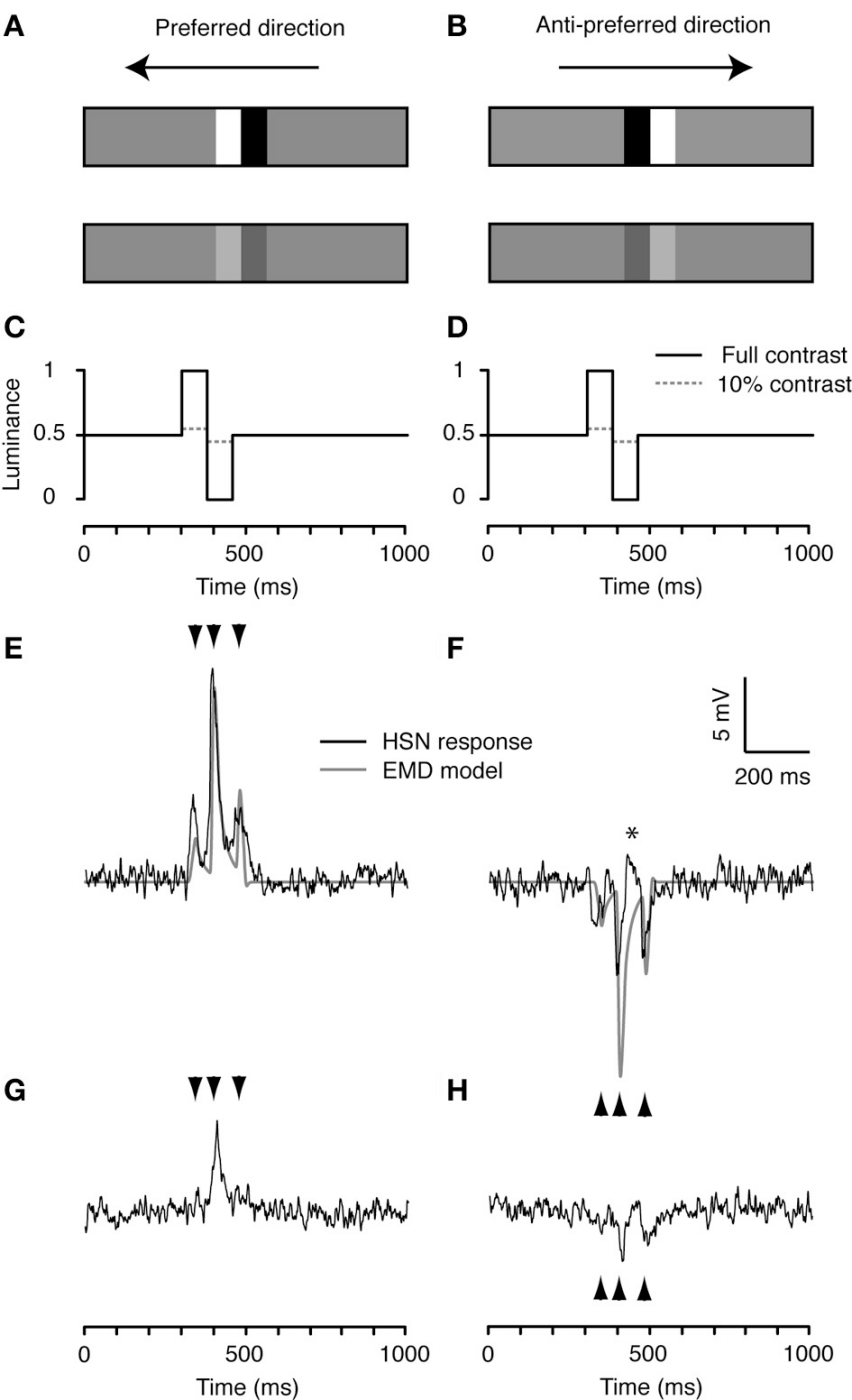
**Doublet stimuli. (A,B)** A combination of two square-wave, white-black luminance steps on mean luminance (gray) background, referred to as a doublet. The doublet is 14° wide and 75° high and has a fundamental row frequency of 0.053 cycles/°, near optimal for hoverfly HS neurons (Straw et al., 2006). We simulated doublet motion at 90°/s with the doublet at either full or 10% contrast in both the preferred **(A)** and anti-preferred direction **(B)**. For display purposes the doublets are not shown at their true contrasts or size. **(C,D)**. Normalized time-luminance graphs as seen at the first edge of the slit-window, i.e., the right hand edge for preferred, right-to-left motion, and the left hand edge for anti-preferred left-to-right motion. Solid black lines represent the full contrast condition, dashed gray lines show the 10% original contrast condition. **(E)** The doublet stimulus produces a characteristic triphasic response from the EMD model in the preferred direction (gray). The neuron's response (black) is also characterized by a triphasic response profile that closely resembles the model output. **(F)** The EMD output (gray) is similar, but inverted, in the anti-preferred direction. The neuron's response is shown in black. The star (^*^) indicates a brief depolarization of the membrane potential. **(G,H)** The neural responses to the low contrast doublet. Arrowheads indicate the timing of the output peaks produced by the model in panels **(E,F)**. Although responses are qualitatively indistinguishable from one recording to the next, absolute response magnitude can vary. To enable accurate comparison of the responses across the six stimulus conditions in Figures 2–4, we show the response to one neuron in which all six conditions were performed, *n* = 20.

We first tested a single cycle of a square-wave (white to black) luminance step on a mean luminance (gray) background, hereafter referred to as a *doublet* (14° wide by the height of the display, Figures 2A,B). This apparently simple stimulus has a fundamental row frequency equivalent to 0.053 cycles/° for a full square wave stimulus, just below the optimum for *Eristalis* (Straw et al., 2006). Using the slit-window mode, we displayed the doublet at both full and 10% contrast, and in both the preferred and anti-preferred direction (Figures 2A,B). We flipped the doublet order before motion in the anti-preferred direction, so that the temporal order of luminance change passing through the slit was always the same (Figures 2C,D).

This stimulus is characterized by three contrast boundaries: gray-to-white, white-to-black, and black-to-gray (Figures 2A,B). The neural response to preferred direction motion is characterized by three peaks, corresponding to the passing of these contrast boundaries through the slit (black, Figure 2E, mean peri-stimulus response 2.93 ± 0.18 mV, *N* = 2, *n* = 40, mean and sem calculated across *n*). The relative magnitude and timing (indicated by arrowheads, Figures 2E,F) of these three peaks are well predicted by the output of a simple computational model for an EMD array to the same stimulus (gray, Figure 2E). Since we use a linear subtraction of the half-units in our basic EMD model, it generates a symmetric output to anti-preferred direction motion (gray, Figure 2F). However, the neural response is not mirror symmetric: First, the three hyperpolarization peaks are much more similar in magnitude. This may be caused by the asymmetry in the subtraction stage of the half-units of the biological EMD (Haag et al., 1999). Second, between the second and third hyperpolarization peak there is a brief, small depolarization of the membrane potential (^*^, Figure 2F). This may reflect the recruitment of voltage-gated sodium conductances within the HS neuron, which boost depolarizing transients (Haag et al., 1997). Nevertheless, overall, the mean peri-stimulus response to anti-preferred direction doublet motion is −1.41 ± 0.16 mV (Figure 2F, *N* = 2, *n* = 40, mean and sem calculated across *n*), 48% of the response to preferred direction motion (Figure 2E).

In response to the doublet presented at 10% contrast, the membrane potential no longer retains an obvious triphasic shape. Instead, the HS neuron gives a single dominant depolarization in the preferred direction (Figure 2G) and two smaller hyperpolarization's in the anti-preferred direction (Figure 2H). In both these cases, the peaks to low-contrast motion correspond in time with the largest peaks observed to high-contrast motion (second arrowhead, Figure 2G; second and third arrowhead, Figure 2H). Despite the 10-fold reduction in stimulus contrast, the responses only rescale by about 1/3 (*R*_pref_: 0.99 ± 0.19 mV, *R*_null_: −0.67 ± 0.23 mV, *N* = 2, *n* = 40, mean and sem calculated across *n*).

### Feature-feature interactions to transient stimuli depend on the temporal order of contrasts

**Figure 3:**
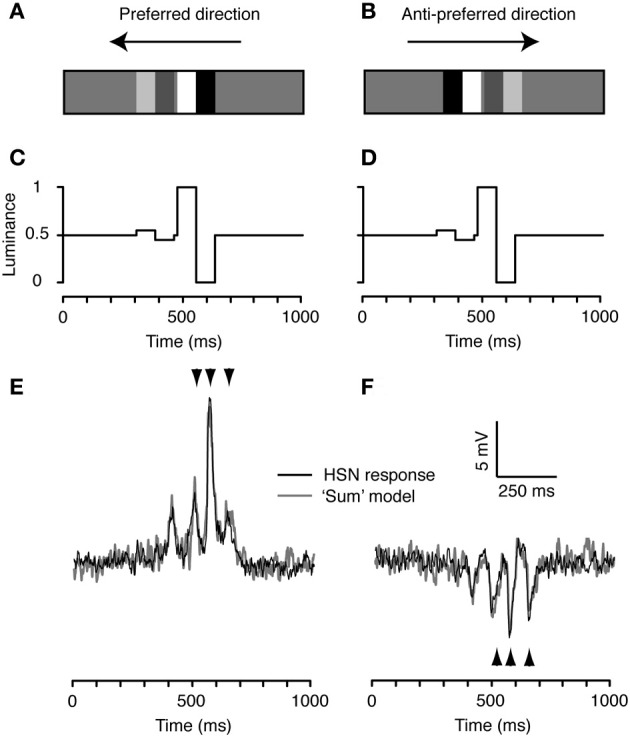
**The increasing contrast feature pair. (A)** We combined the high and low contrast doublets to produce a pair where the low contrast (10%) doublet is followed by the high contrast doublet, referred to as the increasing contrast doublet pair. **(B)** The spatial arrangement is flipped for stimulation in the anti-preferred direction, so that the temporal order of doublet contrasts remains the same. **(C)** Time-luminance trace for motion in the preferred direction. **(D)** Time-luminance trace for motion in the anti-preferred direction. **(E)** Intracellular response of an HS neuron to the increasing contrast pair moving in the preferred direction. The gray line indicates the predicted response based on the linear sum of the response to each individual doublet (as in Figure 2). The arrowheads highlight the timing of the three peaks to the second doublet, predicted from the model output (see Figure 2). **(F)** HS response to the doublet pair moving in the anti-preferred direction. The gray line indicates the linear sum of the response to the individual doublets (see Figure 2). The arrowheads highlight the timing of the three peaks to the second doublet, predicted from the model output. *n* = 20 from the same neuron as shown in Figures 2 and 4.

To investigate the interaction between high and low-contrast features passing the same point in space, we then combined the high and low-contrast doublets from Figure 2. Initially we displayed the two features as an *increasing* contrast pair: the low-contrast feature followed by the high-contrast feature, separated by a 1.2° gap (corresponding to 14 ms at 90°/s; Figures 3A–D). The neuronal response to this increasing contrast pair shows four major peaks (black, Figures 3E,F). These correspond to the peaks generated in response to the single stimuli (Figure 2), as confirmed by the observation that the neural response to the feature pair (black) corresponds well with a simple “model” derived from the linear sum of responses to the individual high and low-contrast stimuli in both the preferred (gray, Figure 3E), and the anti-preferred direction (Figure 3F). Figure 3 displays this result from one neuron, but the same effect was seen in the other HS neuron.

**Figure 4:**
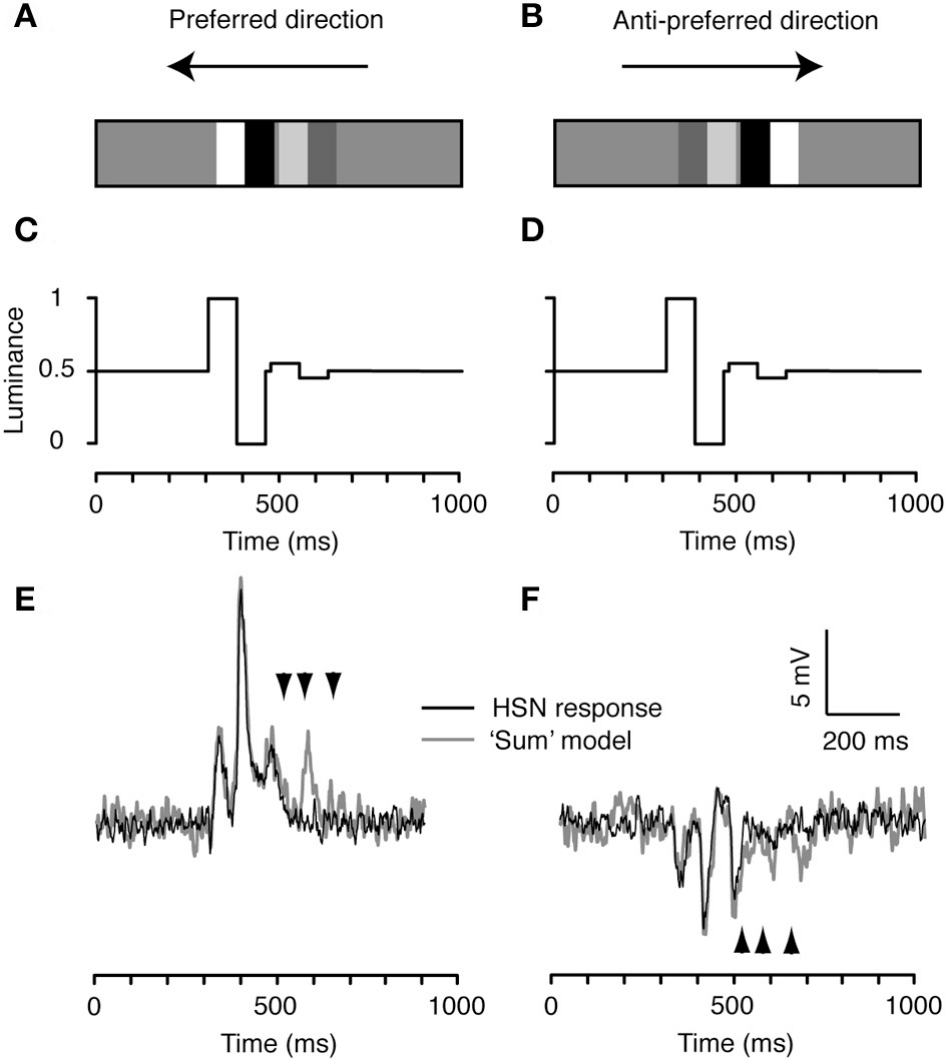
**The decreasing contrast pair. (A)** We combined the high and low contrast doublets to produce an ensemble with the high-contrast doublet preceding the low contrast doublet. **(B)** The spatial arrangement is flipped for stimulation in the anti-preferred direction so that the temporal order of contrast changes remains the same. **(C)** Time-luminance trace for motion in the preferred direction. **(D)** Time-luminance trace for motion in the anti-preferred direction. **(E)** HS response to the decreasing contrast pair moving in the preferred direction. The gray line indicates the predicted response based on the linear sum of the response to each individual doublet (see Figure 2). The arrowheads highlight the timing of the three peaks to the second doublet, predicted from the model output (see Figure 2). **(F)** Intracellular response of an HS neuron to the pair moving in the anti-preferred direction. The gray line indicates the linear sum of the response to the individual doublets (see Figure 2). The arrowheads highlight the timing of the three peaks to the second doublet, predicted from the model output. *n* = 20 from the same neuron as shown in Figures 2 and 3.

We now consider the *decreasing* contrast case, with the doublets rearranged in the opposite order, i.e., with the high-contrast feature followed by the low-contrast doublet (Figure 4). Importantly, this feature pair is identical to the one in Figure 3 with respect to its global spatial frequency power spectrum, luminance, and contrast. It only differs in the temporal order that the different features are seen by local EMDs. Despite this, the neural response differs quite substantially from the prediction based on summing the independently measured doublet responses (compare black and gray, Figures 4E,F). The neuron's response to all three peaks predicted for the low-contrast feature is completely suppressed by the prior passage of the high-contrast feature in both directions (Figures 4E,F). This leads to a 30% net decrease in the mean response in the preferred direction, from 2.1 ± 0.092 mV (*N* = 2, *n* = 40, mean and sem calculated across *n*) to 1.5 ± 0.099 mV (*p* < 0.001; two-tailed *t*-test, significance found in both neurons). It decreases by 15% in the anti-preferred direction, from −1.0 ± 0.12 mV (*N* = 2, *n* = 40, mean and sem calculated across *n*) to −0.85 ± 0.095 mV (no significance found in either neuron). This response reduction presumably reflects local motion adaptation (contrast gain reduction) that is not recruited by the low-contrast feature.

### Global effects of feature-feature interactions within an image

**Figure 5:**
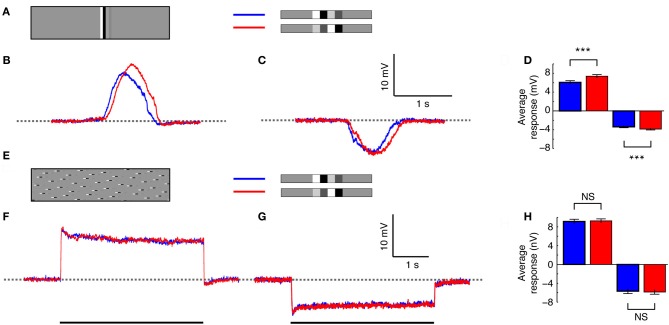
**Re-distribution of the stimulus. (A)** The doublet pairs presented using the whole-screen mode. Blue indicates the decreasing contrast pair, and red the increasing contrast pair. **(B)** Intracellular HS neuron response to the doublets as they pass through the receptive field in the preferred direction (*N* = 8, *n* = 125). **(C)** Intracellular response to the doublets moving in the anti-preferred direction (*N* = 6, *n* = 85). **(D)** The mean response to the doublets moving in the preferred and anti-preferred direction. ^***^Indicates a significant difference (*p* < 0.001, Student's *t*-test). **(E)** The doublet pair broken up into individual pseudo-randomly distributed 1.8° high segments. The stimulus was displayed using the whole-screen mode. The same image was used in all recordings, but due to slight differences in receptive field alignment in respect to the CRT display, the stimulus would never have been identically perceived by two flies. **(F)** Intracellular HS neuron response as the stimulus moves in the preferred direction (*N* = 7, *n* = 67). **(G)** Intracellular HS neuron response as the stimulus moves in the anti-preferred direction (*N* = 5, *n* = 51). **(H)** The mean response to motion in the preferred and anti-preferred direction. NS, no significant difference.

How do the response differences caused by the temporal order of features translate to the neuron's global response (i.e., taking into account of the spatial integration across the receptive field)? To investigate this, we first displayed the same doublet feature pair as in Figures 3 and 4, but in the whole-screen mode (Figure 1A). Responses (Figures 5A–C) now reflect the passage of the features through the underlying HSN receptive field, which has a distinctive frontal “sweet spot” in male flies corresponding to a frontal “bright zone”—a region of enlarged facet lenses that provides locally higher-contrast sensitivity (Straw et al., 2006; Nordström et al., 2008). In both the preferred and anti-preferred direction, the decreasing contrast feature pair (blue, Figures 5B,C) generates a peak response faster than the increasing contrast feature pair (red, Figures 5B,C), likely reflecting the later arrival of the high-contrast feature in the receptive field center (it takes 156 ms for one element of the doublet pair to pass a single point in space at 90°/s). The peak response to the increasing contrast pair (red) is delayed by 131 ms in the preferred direction (Figure 5B) and by 66 ms in the anti-preferred direction (Figure 5C). Confirming our observation in the slit-windowed mode (Figures 3–4), that feature order recruited local adaptation selectively, the mean neural response was significantly larger for the increasing contrast pair: 7.4 ± 0.37 mV compared to 6.1 ± 0.4 mV (*p* < 0.0001, *N* = 8, two-tailed *t*-test, Figure 5D) in the preferred direction, and −3.9 ± 0.2 mV compared to −3.4 ± 0.2 mV (*p* < 0.001, *N* = 6, two-tailed *t*-test, Figure 5D) in the anti-preferred direction.

The temporal order of contrast features clearly retains its substantial effect on the neuron's global response. But does this difference persist if we dissociate the single vertically oriented doublet features across a bigger region of the visual display? To investigate this we separated the doublets into 1.8° high segments (i.e., just larger than the predicted vertical extent of a single EMD), and redistributed these segments pseudo-randomly across the panoramic cylinder. In terms of power spectral density, the resultant image (Figure 5E) is identical along individual 1.8° rows to the image used above (Figure 5A). It differs only in the azimuthal alignment (i.e., local phase) of the doublet pairs between rows. The grossly different response profile (Figures 5F,G) compared to Figures 5B,C highlights an important difference between these stimuli: Because the doublets are spread out, many local features are already present within the most sensitive parts of the receptive field at the commencement of image motion, resulting in a sharp initial response transient, which then decays to a steady-state within a couple of seconds (Figures 5F,G).

Surprisingly, however, this image manipulation reveals no significant difference due to the order of doublet feature pairs (Figures 5F–H). For the increasing contrast pair moving in the preferred direction mean responses were 9.3 ± 0.46 mV, compared to 9.1 ± 0.52 mV for the decreasing contrast pair (Figures 5F,H, *N* = 7). In the anti-preferred direction mean responses were −5.9 ± 0.53 mV for the increasing contrast pair, compared with −5.7 ± 0.59 mV for the decreasing contrast pair (Figures 5G,H, *N* = 5).

Thus, when the stimuli were vertically aligned we saw significant response changes between the two doublet pairs (Figures 5A–D). Yet, despite the fact that each local EMD is stimulated by locally similar feature pairs, when the features were split up and distributed across the screen, there was no longer any response difference due to feature order. There are a couple of important differences between the two stimuli: First, the spatially confined stimulus (Figure 5A) sweeps through the receptive field but commences motion from outside it. As a result, the local feature pair never permits the neuron to reach a steady state response (Figures 5B,C). The spread-out stimulus thus has longer to recruit global components of motion adaptation, as evident from the decay in response over time and the pronounced after-potential following preferred direction motion (Figure 5F). Apparently, these slow components of adaptation are independent of the temporal sequence of local stimulation and dependent only on global activity of the HSN neuron. Second, the vertically-aligned stimulus (Figure 5A) stimulates many local EMDs simultaneously as it enters the receptive field and might thus be expected to be a stronger underlying driver of local neural response for adjacent EMDs. The spatially spread out stimulus (Figure 5E) on the other hand results in fewer doublets present within the receptive field at any one instance, and it may well be expected to be a weaker underlying driver of nearby EMDs. Could the different responses to the two contrast pairs be a consequence of either of these two factors?

### Azimuthal distribution of features

**Figure 6:**
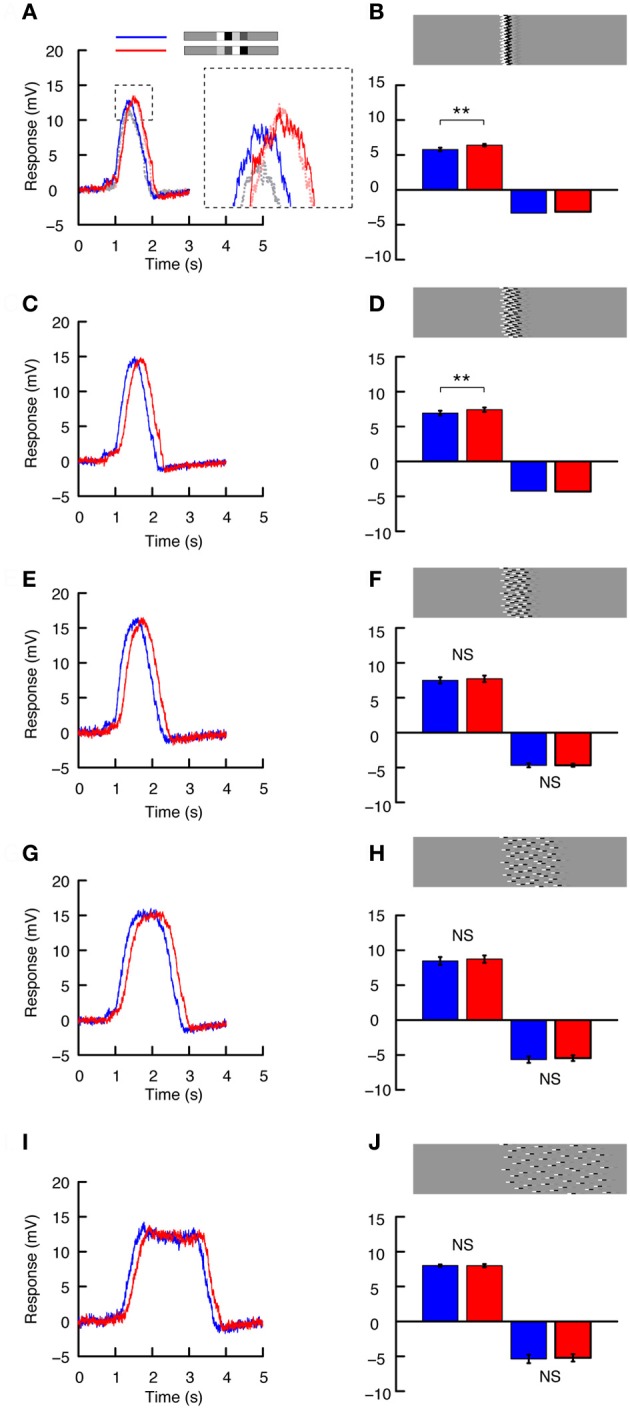
**The azimuthal distribution of features. (A)** Intracellular HS neuron response to preferred direction motion for the image shown in part B, using the whole screen mode (blue = decreasing contrast, red = increasing contrast). The inset highlights the difference between the responses to this image **(B)** and that shown in Figure 5B (dashed gray = decreasing contrast, dashed light red = increasing contrast, data from Figure 5B). **(B)** The doublet pair is broken up into individual 1.8° high segments, which are pseudo-randomly shifted horizontally, so that the maximum horizontal offset is 11° (The absolute spread of the ensembles horizontally is thus, 11° + the doublet pair width, 11° + 30°). The bars show the mean response to motion in the preferred and anti-preferred direction. *N*_pref_= 6, *n* = 65. *N*_null_= 1, *N* = 5 (*t*-test done across repetitions in the single neuron). Stars indicate a significant difference (^**^p < 0.01, Student's *t*-test). **(C)** Response to preferred direction motion of the image shown in **(D)** Once again, the doublet pairs are broken up into individual 1.8° high segments, which are pseudo-randomly shifted such that the maximum horizontal displacement is 22°. The bars show the mean response to motion in the preferred and anti-preferred direction. *N*_pref_= 5, *n* = 55. *N*_null_ = 1, *N* = 5 (*t*-test done across repetitions in the single neuron). Stars indicate a significant difference (^**^p < 0.01, Student's *t*-test). **(E)** As above but for the image shown in **(F)** The doublet pairs are now distributed over 45°. **(F)** The bars show the mean response to motion in the preferred and anti-preferred direction. *N*_pref_= 5, *n* = 55. *N*_null_ = 1, *N* = 5 (*t*-test done across repetitions in the single neuron, errorbar calculated across *n*). NS, no significant difference, Student's *t*-test. **(G)** As above but for the image shown in **H**. **(H)**. The doublet pairs are now distributed over 90°. The bars show the net mean response to motion in the preferred and anti-preferred direction. *N*_pref_ = 5, *n* = 42. *N*_null_ = 2, *n* = 13 (*t*-test done across repetitions independently in the two neurons, errorbar calculated across *n*). NS, no significant difference, Student's *t*-test. **(I)**. As above but for the image shown in **J**. **(J)** The doublet pairs are now distributed over 180°. The bars show the mean response to motion in the preferred and anti-preferred direction. *N*_pref_ = 3, *n* = 27 (*t*-tests done across repetitions independently in the three neurons, errorbar calculated across n). *N*_null_ = 2, *n* = 15 (*t*-tests done across repetitions independently in the two neurons, errorbar calculated across n). NS, no significant difference, Student's *t*-test.

To investigate the relationship between local adaptation (contrast gain reduction) and the degree of alignment of local features, we designed images representing a continuum from being perfectly aligned (as in Figures 5A–D) to fully spread across the display (as in Figures 5E–H). Remarkably, introduction of even a small offset into image rows leads to pronounced differences in the influence of doublet feature order (Figure 6). For example, in Figures 6A,B the maximum offset in vertically neighboring rows is only 11°. Although there is still a difference in the peak responses produced by the two contrast pairs (compare blue and red, Figures 6A, *N* = 6), this difference is substantially smaller than that observed for the perfectly vertically aligned stimulus (dashed data, inset, Figure 6A). Importantly, the absolute response to the increasing contrast pair is similar to that in Figure 5 (red, Figure 6A). But the response to the decreasing contrast pair is now substantially larger (compare dashed gray and blue lines, inset, Figure 6A). Quantitative analysis reveals that the mean neural response is different between the doublet pairs in the preferred direction (Figure 6A, *N* = 6, *p* < 0.01). This small image manipulation results in no significant difference in the anti-preferred direction (Figure 6B), but this was only confirmed in one neuron (*N* = 5).

As we spread the features out further (with segments varied by up to 22°), the overall neural response *increases* slightly for both the decreasing and increasing contrast conditions (Figures 6C,D). However, the difference due to doublet feature order is even smaller. Preferred direction motion still generated a weaker response for the decreasing contrast condition (Figures 6C,D, *N* = 5, *p* < 0.01), while in the anti-preferred direction there is no response difference (Figure 6D, *N* = 1, significance tested across *N* = 5). Dispersing the doublet pairs further across the panorama produced even larger net neural responses (Figures 6E–H). However, the two different contrast pairs no longer generated different neural responses in either direction of motion. In the final example, we spread the doublet pairs out over more than half the panorama (Figure 6J). In this case the neural response clearly reaches steady-state. The neural response has become weaker, and there is no magnitude difference between the two contrast pairs (Figures 6I,J).

It thus appears that only a slight horizontal misalignment of doublets drastically alters the influence of local gain reduction recruited by a high-contrast feature passing each location before a lower-contrast feature. We also note that the response difference between the low- and high-contrast doublet pairs disappears (Figure 6F) before the apparent longer-duration “steady-state” responses (Figure 6I), so this effect appears to be independent of recruitment of slow global adaptation.

### Simultaneous stimulation of neighbouring local motion sensitive elements recruits a powerful reduction of motion detector gain for subsequent features

**Figure 7:**
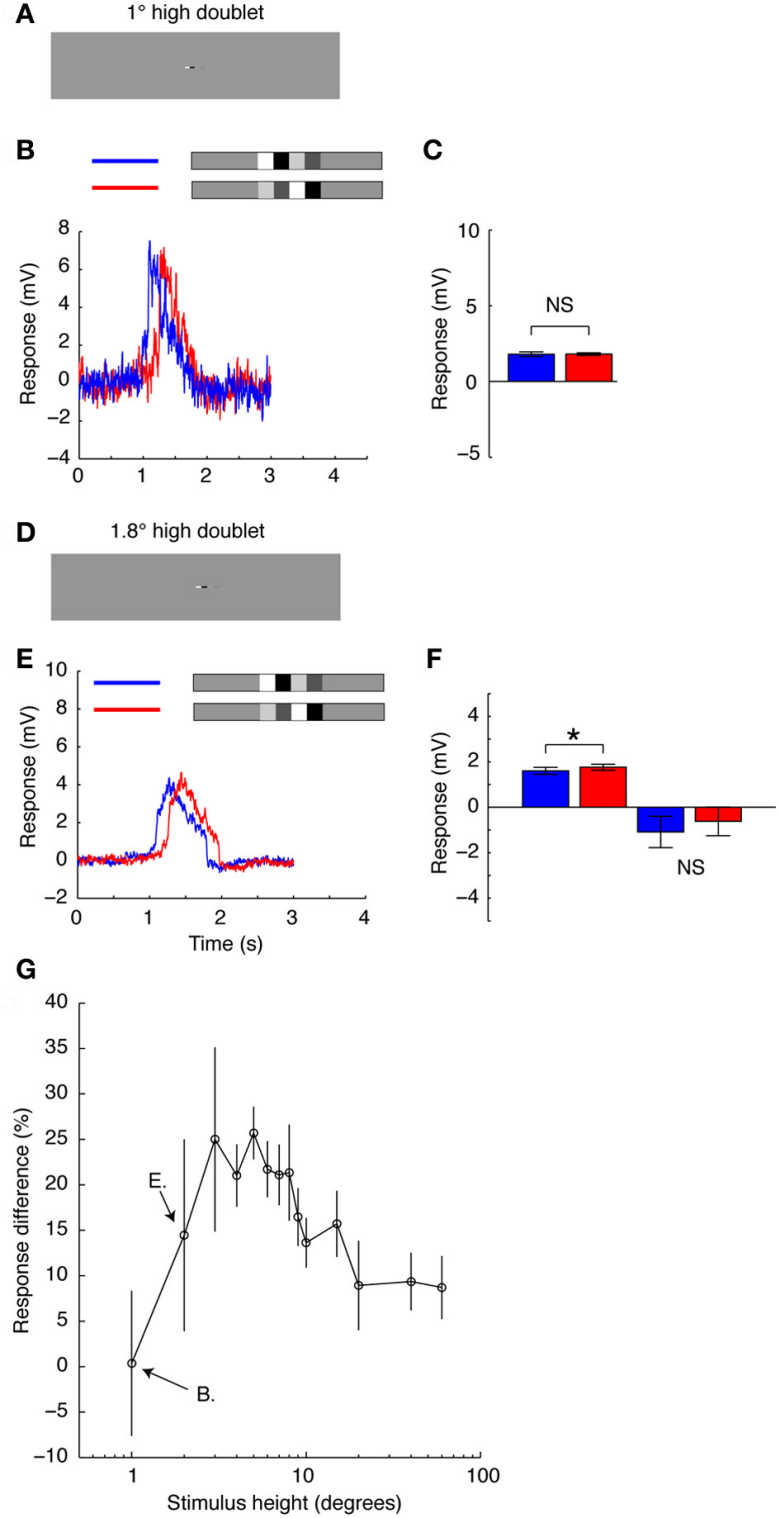
**The vertical extent of a small stimulus. (A)** The doublet pairs presented using the whole-screen mode. The doublet pair has the same width as before, but is now only 1° high, just below the size of an individual ommatidium. **(B)** Intracellular HS neuron response to the doublet pair shown in **A**, as it passes through the receptive field in the preferred direction (blue = decreasing contrast, red = increasing contrast). **(C)** The mean response to the doublets as shown in part B. *N* = 1, *n* = 9 (*t*-test done across repetitions in the single neuron, errorbar calculated across *n*). NS, no significant difference (Student's *t*-test). **(D)** The doublet is now 1.8° high. **(E)** Intracellular HS neuron response to the doublets as they pass through the receptive field in the preferred direction. **(F)** The mean response to the doublets moving in the preferred and anti-preferred direction. *N*_pref_ = 4, *n* = 135. *N*_null_ = 1, *n* = 20 (*t*-test done across repetitions in the single neuron, errorbar calculated across *n*). ^*^Indicates a significant difference (*p* < 0.05, Student's *t*-test). NS, no significant difference (Student's *t*-test). **(G)** The average response difference between the increasing and decreasing contrast pairs as a function of their vertical extent. A positive difference indicates that the response to the increasing contrast ensemble is larger. *N* = 1, *N* = 8 (errorbars calculated across *n*).

To further investigate the hypothesis that the change in neural response observed when the feature pairs are vertically aligned results from the interactions of simultaneously activated neighboring EMDs, we limited the vertical extent of the whole stimulus (Figure 7). If the response reduction is the result of the simultaneous activation of vertically aligned, local EMDs feeding into the HS neuron, the reduction should disappear by limiting the stimulus height to just one row of local EMDs. When we limited the height of the stimulus to 1° (Figure 7A), approximately the same size as the receptive field of an individual ommatidium in *Eristalis tenax* (Straw et al., 2006), we see no change in mean response between the two doublet pairs (Figures 7B,C). The mean response was 1.8 ± 0.091 mV (*N* = 1, *n* = 9, mean and sem calculated across *n*) for the increasing contrast pair and 1.8 ± 0.15 mV (*N* = 1, *n* = 9, mean and sem calculated across *n*) for the decreasing contrast pair moving in the preferred direction. However, as soon as we extend the stimulus to stretch across more than one ommatidium, just 1.8° (Figures 7D–F) the increasing contrast pair produces a stronger response than its counterpart just as observed in the earlier experiments (Figures 5–6). For the increasing contrast pair, the mean responses were 1.76 ± 0.19 mV compared with 1.60 ± 0.19 mV for the decreasing contrast pair in the preferred direction (*N* = 4, *p* < 0.001; Figure 7F). In the anti-preferred direction, the response change was in the opposite direction. However, the variability was large and not significant (Figure 7F, *N* = 1, *n* = 16, mean and sem calculated across *n*).

In a long duration recording from a single neuron we were able to further quantify this effect across numerous stimulus heights to show that even for relatively small increases in stimulus height, the increasing contrast pair produces up to a 25% stronger response than the decreasing contrast pair (Figure 7G). The maximum difference in mean response between the two doublet pairs is reached at stimulus heights between 3 and 8° (Figure 7G). After the stimulus exceeds 10°, the difference between the two stimulus pairs gets gradually smaller, in part reflecting saturation of the responses that transiently exceed 12 mV in both cases for full height stimuli (see Figure 5). These data support the hypothesis observed in Figures 5–6 that simultaneous stimulation of vertically aligned EMDs contributes to the observed response difference to the increasing and decreasing contrast doublet pairs.

## Discussion

### Locally acting response-gain reduction

In this paper we show that the temporal order of high- and low-contrast features can strongly influence the global response of fly LPTCs under some, but not all conditions. If vertically aligned doublet features are shown as an increasing contrast pair, the neural response can be predicted by the linear sum of the responses to the individual doublets (Figure 3). However, when the features are presented as a decreasing contrast pair, the responses were no longer consistent with the linear sums of the individual components (Figure 4). Instead, strong local motion adaptation was recruited by the high-contrast doublet and inhibited the responses to subsequent low-contrast features. It is thus clear that even transient stimulation by high-contrast features induces sufficient sensitivity reduction to reduce subsequent responses to other features.

Our data further show that the sensitivity reduction by high-contrast features is recruited locally (Figures 5–7). Motion adaptation has previously been shown to consist of different physiological components (Harris et al., 2000; Kohn and Movshon, 2003) where the contrast gain reduction is local (Nordström and O'Carroll, 2009) and quickest to appear (Nordström et al., 2011). We thus find it likely that the effects observed here act on the neuron's contrast gain. Nevertheless, even if the observed adaptation is recruited locally, the reduction in response gain has its strongest effect by simultaneous activation of at least neighboring local motion sensitive elements (Figures 5–7) and is thus severely disrupted by breaking an elongated feature into more localized segments. We thus conclude that whereas earlier work showed that adaptive gain reduction is recruited locally (Nordström and O'Carroll, 2009), within the EMDs themselves (De Haan et al., 2012; Rien et al., 2012), this contrast gain reduction is somehow also dependent on the more global structure of the features within a scene and is much more pronounced for coherent, vertically aligned stimuli (Figure 5).

It is particularly noteworthy that when the stimulus was confined to a vertical extent smaller than one EMD, no local adaptation was recruited by the high-contrast doublet (Figures 7A–C). However, extension to just under double this height was sufficient to recruit powerful local motion adaptation (Figures 7D–F). At 1.8° high, this stimulus almost certainly simultaneously stimulates more than a single EMD as it passes through the receptive field. Therefore, it is likely that the response reduction to the decreasing contrast feature pair is resulting from coupled interactions between vertically neighboring EMDs (i.e., orthogonal to the direction of motion). This observation is supported by early work on the input elements to LPTCs, which showed that these are likely to be orientation selective, with a preference for vertically aligned stimuli (Srinivasan and Dvorak, 1980). The spatial pre-filters were shown to have a Gaussian half-width of 2.2° (Srinivasan and Dvorak, 1980) to 2.6° (Arnett, 1972) along the vertical axis. These studies further showed that the spatial pre-filters are flanked by horizontally neighboring inhibitory surrounds. Such lateral inhibition should sharpen the selectivity for vertically aligned features, since even slight misalignment in the horizontal domain would induce lateral inhibition. Lateral inhibition at the spatial pre-processing stages is likely to be provided by the lamina monopolar cell L4 (Fischbach and Dittrich, 1989).

Since the spatial pre-filters are likely to be vertically oriented (Arnett, 1972; Srinivasan and Dvorak, 1980), this could provide a further explanation for our data in Figure 5. In the vertically aligned stimulus, fewer inputs are activated, but each unit is activated strongly (since the stimulus will extend across its entire vertical axis). In the spread-out stimulus, however, more inputs are activated, but each unit receives less activation, since the vertical extent of the receptive field is larger than the 1.8° height of the stimulus. The input pre-filters are spatially pooled in the HS cell, where the summed responses may end up being similar. However, since contrast gain reduction is likely to be generated early within the motion processing pathway (Nordström and O'Carroll, 2009; Nordström et al., 2011; De Haan et al., 2012), likely just down-stream of these very inputs, the contrast gain reduction will be most strongly recruited when the stimulus is vertically aligned (Figure 5A), and therefore the presentation order of the features has its strongest effect in the vertically aligned stimuli.

In mammalian V1 and also auditory neurons local responses have been shown to be scaled by adaptive processes based on local surround excitation (Schwartz and Simoncelli, 2001). In these examples local adaptation can be modeled by a divisive feedback of surround activity, therefore normalizing local neural response based on its surround. Such adaptive normalization strategies have the advantage over linear filters in that they rescale neural response and maximize coding range for the prevailing stimulus. Such local adaptive rescaling could be particularly advantageous in the encoding of natural image motion, as natural scenes have local structures and contrasts that are highly erratic from one location to the next (Frazor and Geisler, 2006; Rieke and Rudd, 2009).

### Modelling of responses

We here used a basic EMD model to show that the three response transients to the windowed stimulus are predicted from the basic computations of motion. Even if more elaborate models might be able to recapture some of our observations from physiology, recent work has highlighted how much remains to be known about the precise processing that takes place in the EMD (see e.g., the conflicting results in Clark et al., 2011; Eichner et al., 2011). Nevertheless, it is likely that ON and OFF stimuli are separated early in the visual pathway, via the lamina monopolar cells L1 and L2, respectively (Joesch et al., 2010) and then transmitted to T4 and T5 (Schnell et al., 2012). This separation suggests that the responses that we recorded here, to bright and dark contrast increments, respectively (Figures 2A–D), are likely processed via separate pathways. Importantly, while the L1 and L2 pathways may provide separate inputs to their postsynaptic (T4, T5) targets (Joesch et al., 2010; Schnell et al., 2012), the lamina cells themselves respond to (and adapt to) both signs of contrast. Since earlier work already showed that local flicker stimuli (which recruit contrast adaptation in lamina cells) are at best weak drivers of the pronounced motion-dependent contrast gain reduction for HS neurons (Harris et al., 2000) it is tempting to propose that the gain reduction we observe occurs in the half-wave rectifying subunits of the EMD. This hypothesis could be tested in HS neurons using an ON-ON high-contrast feature, followed by an OFF-OFF low-contrast feature, the responses to which should then be unaffected by the prior passage by a bright stimulus. Differential processing of ON and OFF input is supported by medulla work from flies (Wiederman et al., 2008) and other insects (O'Carroll et al., 1992), as well as by the finding that a light-dark transition causes a larger response transient than a dark-light transition (Jansonius and van Hateren, 1991).

Future modeling may also be able to deduce how large influence dendritic gain control and spatial saturation (see e.g., Borst and Haag, 2002) have on the response profiles that we recorded to vertically aligned features (Figure 5). Importantly, to be able to model the global response properties correctly, we need a compartmental model of the hoverfly HS neurons. Currently, physiologically relevant compartment models exist for blowfly LPTCs (see e.g., Borst and Weber, 2011), but not for hoverflies. Compartment models would be able to more precisely compute how the adaptation and saturation components spread across the dendritic tree in different dimensions, and determine the influence this gain control would have on the responses to the different stimuli displayed here.

Our basic EMD model (gray, Figures 3E,F), despite containing biomimetic spatio-temporal inputs, does not recapture several known properties of dipteran HS neurons. For example, in the physiological responses we saw a “rebound” depolarization (^*^, Figure 3F) following a strong hyperpolarizing transient in response to anti-preferred direction motion. A similar effect is not evident in the preferred direction. This asymmetry likely reflects the recruitment of voltage-gated sodium conductances (Haag and Borst, 1996). Sodium conductances are associated with the neuron-specific, monophasic “spikelets” found in dipteran HS and VS neurons. The spikelets start to resemble typical discrete action potentials if the neuron is hyperpolarized (Haag and Borst, 1998), and are particularly elevated following anti-preferred direction stimulation (Nordström and O'Carroll, 2009). The role of spikelets in neural coding is still under debate (see e.g., Haag and Borst, 1996, 2008; Kretzberg et al., 2001; Beckers et al., 2009), which is why we chose to spike-filter our data to decrease their influence. However, since spikelets are highly irregular it is impossible to remove all of them, particularly when the neuron is strongly depolarized. Since spikelets are monophasic the unfiltered spikelets will subsequently predominantly skew the measured membrane potential toward depolarizing values, and thus contribute to the asymmetry of response between preferred and anti-preferred direction motion that is not captured by our model. Our model also had no output saturation, which would tend to dampen large transients in either direction. Ironically, such saturation would act in opposition to the depolarizing transient enhancement for preferred direction induced by active conductances. This may explain the much better fit qualitatively captured by our parsimonious model for preferred direction motion (Figure 2E).

### White noise techniques

In this paper we chose to use experimenter-designed stimuli to quantify the effect of the spatio-temporal distribution of features. Our stimuli are thus Cartesian, local and relevant for the type of features that might constitute a subset of natural scenes, yet neither random nor truly natural. They have the advantage of giving the experimenter more control over their statistics than possible with naturalistic stimuli. Another potential option would have been to use white noise stimuli for deducing the neural sensitivity (Ringach and Shapley, 2004) and potentially even to investigate the spatiotemporal receptive field—i.e., the degree to which stimulation at one location is dependent on simultaneous or prior stimulation at adjacent regions (van Kleef et al., 2010). 2D white noise techniques (e.g., m-sequence stimuli, Ringach and Shapley, 2004) may be an interesting approach to apply in future work, particularly to investigate the vertical interactions that we revealed between local EMDs. Complicating this approach, however, white-noise methods assume that the spatio-temporal receptive fields are time invariant, i.e., that the response kernel extracted from a white noise stimulus can be utilized to predict the neuron's response to any type of other stimulus. Since most higher-order visual neurons adapt, the response properties and the computation of motion depend heavily on the neuron's stimulus history (for thorough discussion of this, see van Kleef et al., 2010). Our main finding is that it is precisely such a potent dynamic non-linearity (i.e., local motion adaptation) that affects responses to subsequent low-contrast stimuli. Any future application of white-noise techniques to these questions would thus need very sparse stimulus sequences (van Kleef et al., 2010).

### Role of local adaptive gain reduction in the encoding of image motion

What role might a mechanism influencing the gain of local motion sensitive elements play in the encoding of motion in the natural environment? Accurately interpreting natural motion is an extremely challenging task for visual systems of any kind. Natural scenes often contain highly variable structures and contrasts (Tolhurst et al., 1992), both parameters that are known to modulate the response of biological motion detectors (see Borst and Egelhaaf, 1989; Borst et al., 2010).

The EMD is generally accepted to underlie local motion computation in insects and other animals (Borst and Euler, 2011). However, it generates ambiguous estimates of image velocities because it is sensitive to e.g., image contrast and spatial structure (Dror et al., 2001; Shoemaker et al., 2005; Straw et al., 2008). Although many behavioral and neurophysiological response properties corroborate the predictions of the EMD (Borst et al., 2010), substantial evidence suggests that insects use apparent retinal velocities to control navigation (Srinivasan et al., 1991; Esch and Burns, 1996; Baird et al., 2005; Grah et al., 2005). Interestingly, when natural images are used as stimuli, LPTCs actually respond independent of contrast and spatial structure, and encode image velocity robustly (Straw et al., 2008; Barnett et al., 2010). This has been hard to reconcile with LPTC response characteristics to experimenter-defined stimuli, such as sinusoidal gratings, and with the outputs of EMDs (Dror et al., 2001; Shoemaker et al., 2005; Straw et al., 2008). Our recent work found it unlikely that static compressive non-linearities such as response saturation alone could explain the robust encoding of image velocity (Barnett et al., 2010; O'Carroll et al., 2011). It is, however, possible that local adaptive gain control of the type shown here might play an important role in normalizing local neuronal outputs based on the neighboring conditions in space and time.

We here showed a particularly prominent importance of vertically oriented visual features in the encoding of motion by fly LPTCs. Here we used experimenter-designed stimuli, but previous work supports the suggestion that vertically oriented features in natural scenes also strongly influence responses of fly HS neurons (Liang et al., 2008; Meyer et al., 2011; O'Carroll et al., 2011). In particular, it is difficult for LPTCs to code velocity reliably in natural scenes that lack vertical features (Barnett et al., 2010). In behavioral experiments flies and other insects orient toward vertically oriented features. This behavior is robust not only in walking (Robie et al., 2010), but also during tethered flight (Götz, 1987) and free flight (Maimon et al., 2008). When vertically oriented features are reduced in height, free flying *Drosophila* are no longer attracted to them, but instead avoid them (Maimon et al., 2008). The physiological and behavioral preference for vertical contours is intriguing considering the spatial structure of natural scenes. In a large FFT analysis of 12,000 scenes it was shown that vertical and horizontal contours dominate over contrast borders of other orientations (Torralba and Oliva, 2003). Taken together this highlights the importance of vertically oriented high-contrast features in the robust encoding of visual motion, and highlights the neurocomputational match between the visual input and its sensors (Girshick et al., 2011).

### Conflict of interest statement

The authors declare that the research was conducted in the absence of any commercial or financial relationships that could be construed as a potential conflict of interest.

## Abbreviations

EMD: elementary motion detector
HS: horizontal system
LPTC: lobula plate tangential cell.
